# Pain Catastrophizing Correlates with Early Mild Traumatic Brain Injury Outcome

**DOI:** 10.1155/2016/2825856

**Published:** 2016-03-02

**Authors:** Geneviève Chaput, Susanne P. Lajoie, Laura M. Naismith, Gilles Lavigne

**Affiliations:** ^1^Department of Cancer Support and Palliative Medicine, McGill University Health Centre, Montreal, QC, Canada H4A 3J1; ^2^Department of Educational and Counselling Psychology, McGill University, Montreal, QC, Canada H3A 1Y2; ^3^HoPingKong Centre for Excellence in Education and Practice, University Health Network, Toronto, ON, Canada M5T 2S8; ^4^Department of Dentistry, Université de Montréal, Montreal, QC, Canada H3C 3J7

## Abstract

*Background.* Identifying which patients are most likely to be at risk of chronic pain and other postconcussion symptoms following mild traumatic brain injury (MTBI) is a difficult clinical challenge.* Objectives.* To examine the relationship between pain catastrophizing, defined as the exaggerated negative appraisal of a pain experience, and early MTBI outcome.* Methods.* This cross-sectional design included 58 patients diagnosed with a MTBI. In addition to medical chart review, postconcussion symptoms were assessed by self-report at 1 month (Time 1) and 8 weeks (Time 2) after MTBI. Pain severity, psychological distress, level of functionality, and pain catastrophizing were measured by self-report at Time 2.* Results.* The pain catastrophizing subscales of rumination, magnification, and helplessness were significantly correlated with pain severity (*r* = .31 to .44), number of postconcussion symptoms reported (*r* = .35 to .45), psychological distress (*r* = .57 to .67), and level of functionality (*r* = −.43 to −.29). Pain catastrophizing scores were significantly higher for patients deemed to be at high risk of postconcussion syndrome (6 or more symptoms reported at both Time 1 and Time 2).* Conclusions.* Higher levels of pain catastrophizing were related to adverse early MTBI outcomes. The early detection of pain catastrophizing may facilitate goal-oriented interventions to prevent or minimize the development of chronic pain and other postconcussion symptoms.

## 1. Introduction

Traumatic brain injuries (TBIs) constitute a leading cause of disability around the world [1–3]. Between 75% and 90% of TBIs are typically classified as mild traumatic brain injuries (MTBIs, 4). Symptoms reported during the acute phase (within days) following MTBI are generally categorized as physical, psychological, or neurocognitive in nature [4–6]. Although physical symptoms are typically reported in the acute phase following injury, discrepancies remain regarding the nature and prevalence of other symptoms after MTBI [7–9]. Previous studies suggest that up to 80% of patients present at least one symptom in the acute period, and close to 50% of patients report one or more persisting symptoms at 3 months following MTBI [10, 11].

Pain is a significant problem following TBI and more particularly after a milder traumatic brain injury [12]. A wide array of factors have been proposed to explain the greater pain experience in MTBI in comparison to more severe brain injuries, including shorter recovery period secondary to prompter return to regular activities [13], as well as increased awareness and perception of pain resulting from lesser severity or absence of loss of consciousness and posttraumatic amnesia following MTBI [14]. While pain usually resolves within the first 3 months [15], persistent symptoms including headaches, sleep disturbances, and fatigue have been reported in 5% to 15% of patients months to years following the head trauma [4, 16].

Identifying which patients are most likely to be at risk of chronic pain and other postconcussion symptoms is a difficult clinical challenge [17]. Recent findings have provided important insights into the clinical [18, 19], neurocognitive [20], and rehabilitation [21] factors that influence MTBI outcome. Factors associated with postconcussion symptoms at 1 month included presence of a skull fracture, dizziness, and headache [22], while level of education, lack of nausea or vomiting, and low levels of pain were associated with full return to work by 6 months [17]. Despite this progress, understanding the psychological processes that influence MTBI outcome represents an important and underrepresented area for further research [23].

The objective of the present study was to examine the role of pain coping styles in MTBI outcome. Pain catastrophizing, defined as an exaggerated appraisal of the negative components of an actual or anticipated pain experience [24, 25], is a coping style characterized by three catastrophizing dimensions: rumination, magnification, and helplessness [25]. Literature findings have consistently demonstrated relationships between heightened pain and catastrophizing in both healthy asymptomatic individuals [25–27] and various patient groups [28, 29]. Recent findings have demonstrated associations between catastrophizing and abnormal changes in brain morphology and function amongst migraine patients [30]. Catastrophizing has also been associated with illness behaviors such as frequency of visits to healthcare professionals and hospital stays [31], as well as use of both hospital administered and over-the-counter primary medications [32, 33]. A relationship between catastrophizing and adverse clinical outcome has also been illustrated in patients with soft-tissue injuries [34] and fibromyalgia [35]. With respect to MTBI outcome, we hypothesized that higher levels of pain catastrophizing would be associated with greater severity of self-reported pain, greater number of postconcussion symptoms, increased psychological distress, decreased level of functionality, and higher risk of postconcussion syndrome. We conducted a cross-sectional cohort study to test these hypotheses.

## 2. Methods

### 2.1. Participants

A sample of 58 participants was recruited as part of a larger-scale study assessing sleep patterns and genotypical components of MTBI patients. Participants were recruited from those seen at the emergency department (ED) of an accredited specialized trauma centre in Montreal, Canada, and diagnosed with a MTBI. MTBI diagnosis was established by a certified neurosurgeon based on the following Task Force criteria: state of consciousness, presence of posttraumatic amnesia, and Glasgow Coma Scale (GCS) score [36]. Inclusion criteria consisted of any patient seen at the hospital for a day or more and whose posttrauma symptoms were not due to alcohol intoxication, illegal substance or medication, or other injuries (systemic or facial lesions, intubations). Exclusion criteria included age <18 years, language barrier, known history of mental retardation, previous history of TBI, previous diagnosis of dementia or learning disability impairing the patient's cognitive and reasoning processes, or current coexisting neurological or psychiatric illness.

A trauma research nurse recruited participants between January 2006 and August 2010. During this time period, 2733 patients were seen in the ED and diagnosed with a MTBI. Of these, 128 were recruited for study participation and 80 consented to participate (63%). Twenty-two participants were later excluded because they did not meet the inclusion criteria, voluntarily withdrew from the research protocol, or provided incomplete study data. Institutional ethics approval was granted for this study and all participants provided written consent.

### 2.2. Measures

#### 2.2.1. Patient Characteristics and Medical History

Patient charts were reviewed for previous medical and surgical history and previous history of traumatic brain injury. A systematic search targeted the following illnesses: sleep disturbances, chronic fatigue syndrome, psychiatric illness, and/or neurological impairment. Current medication intake, habits (smoking, alcohol, and drug use), known allergies, data pertaining to the traumatic injury leading to the MTBI, and medical and/or surgical treatment received during the hospital stay were also recorded.

#### 2.2.2. Pain Catastrophizing

The 13-item pain catastrophizing (PC) scale was used to measure pain catastrophizing [26]. The scale asks participants to rate the frequency of catastrophizing thoughts or feelings (0 = not at all; 4 = all the time). The 13 catastrophizing items are further divided into 3 dimensions: magnification (3 items), rumination (4 items), and helplessness (6 items), a structure which has been replicated in other studies [37]. Magnification describes an excessive focus on the negative aspects of a pain experience and expectancies for a negative outcome (“I become afraid that the pain will get worse”); rumination refers to excessive worry and an inability to inhibit pain-related thoughts (“I cannot seem to keep it out of my mind”); and helplessness refers to a perceived inability to cope with a pain experience (“It's awful and I feel that it overwhelms me”). Internal consistency estimates range from *α* = .66 for magnification to *α* = .87 for total PC [26].

#### 2.2.3. Postconcussion Symptoms

The 16-item Rivermead Postconcussion Symptoms Questionnaire (RPQ) was used to measure postconcussion symptoms [38]. This scale asks participants to rate the degree to which postconcussion symptoms are more problematic in comparison to premorbid levels (0 = not experienced at all; 4 = severe). Specific symptoms targeted include headaches, dizziness, nausea, noise sensitivity, sleep disturbance, fatigue, irritability, feeling depressed, frustration/impatience, forgetfulness, poor concentration, taking longer time to think, blurred vision, light sensitivity, double vision, and restlessness. The RPQ Headache item was used as a measure of headache pain.

#### 2.2.4. Psychological Distress and Level of Functionality

The Multidimensional Pain Inventory (MPI) scale was used to measure behavioral, cognitive, and emotional dimensions of living with pain [39]. The MPI scale consists of three sections (52 items in total), which ask participants to rate how much their pain affects their lives, how others react to their communications of pain, and the frequency with which they participate in common daily activities. The Pain Severity subscale (3 items) was used as a measure of general pain severity (“How much suffering do you experience because of your pain?”); the Affective Distress subscale (3 items) was used as a measure of psychological distress (“During the past week, how irritable have you been?”); and the General Activities subscale (18 items) was used as a measure of level of functionality, based on frequency of participation in household chores, outdoor work, activities away from home, and social activities. Internal consistency estimates range from *α* = .70 to .90 for all MPI scales [39].

#### 2.2.5. Timing

A trauma research nurse administered the RPQ at 1 month after MTBI (Time 1, mean = 33 days, response rate = 97%). Participants completed the MPI scale, the PC scale, and a second RPQ at home within 6–8 weeks of their diagnosis and returned their completed questionnaires to the hospital by mail (Time 2, mean = 55 days). Response rates ranged from 83% for the second RPQ to 100% for the PC scale.

### 2.3. Data Analysis

Patient characteristics and medical history were analyzed descriptively. Relationships between pain catastrophizing and other Time 2 measures (pain severity, postconcussion symptoms, psychological distress, and level of functionality) were assessed using Pearson's correlations (*r*). To assess whether pain catastrophizing may be a risk factor for the development of postconcussion syndrome, we first classified participants as being at either high or low risk for this condition. Postconcussion syndrome has been previously defined as the persistence of 3 or more symptoms following MTBI [40]. As this definition lacks specificity to the TBI population [41], we established a novel, more conservative definition. Participants who reported 6 or more RPQ symptoms at a level of 2, 3, or 4 at both Time 1 and Time 2 were categorized as “High Risk” for developing postconcussion syndrome, while participants who reported 5 or less symptoms at either time point were categorized as “Low Risk” for this condition. Student *t*-tests were used to compare the mean subscale and overall catastrophizing scores between High Risk and Low Risk groups. For all statistical tests, significance was defined as *p* < .05.

## 3. Results

### 3.1. Demographic and Clinical Characteristics

Patient characteristics are summarized in Table 1. The mean patient age was 39.6 years (min: 19 to max: 63) and 72% (42) were men. Amnesia and loss of consciousness were confirmed in 17% and 66% of cases, respectively. Mean GCS scores recorded at initial evaluation (at scene of trauma) and at ED assessment were 13.9/15 and 14.5/15, respectively. Mean length of stay at hospital was 3.88 days (min: 1 to max: 25). The most frequent mechanisms of injury were motor vehicle accidents (35%) and accidental falls (26%). In 17% of cases, the injury occurred in the patient's work setting. Alcohol intoxication was confirmed or clinically suspected in 12% of cases.

In addition to the MTBI, 36% of patients sustained one or more concomitant injuries, including fracture (50%), contusion (14%), or laceration (19%). Thoracic (69%) and facial (45%) areas were the most common fracture locations. Pulmonary, myocardial, and thoracic contusions accounted for the majority of contusions recorded, in 7%, 5%, and 3.5% of cases, respectively. Sustained lacerations were superficial in nature and localized in head and face locations in the majority of cases (73%).

A CT-scan was performed on 88% of patients. Of these, 54% revealed abnormal findings, with acute subarachnoid hemorrhage (19%), hemorrhagic contusions (16%), and punctiform hematoma (13%) being the most frequent radiological diagnoses. The majority of patients (83%) received one or more analgesics in hospital. Final prognosis was deemed favorable by a multidisciplinary team assessment in 90% of patients, with the remainder being classified as unfavorable (4%) or unspecified (7%). Following hospital discharge, most patients were sent home (95%), while the remainder were transferred to a rehabilitation facility.

### 3.2. Pain Catastrophizing and Pain Severity

Headache pain (RPQ Headache) at Time 2 was associated with PC Magnification (*r* = .35, *p* = .02). General pain at Time 2 (MPI Pain Severity) was associated with PC Rumination (*r* = .33, *p* = .02), PC Magnification (*r* = .31, *p* = .03), PC Helplessness (*r* = .44, *p* < .01), and overall PC (*r* = .39, *p* < .01).

### 3.3. Pain Catastrophizing and Postconcussion Symptoms

The most frequently reported RPQ postconcussion symptoms at Time 1 were fatigue (70%), sleep disturbances (66%), dizziness (52%), headaches (50%), and reduced ability to concentrate (50%). Symptoms reported at Time 2 included fatigue (69%), sleep disturbances (63%), reduced ability to concentrate (63%), and dizziness (60%) (Table 2). RPQ assessments at Time 1 and Time 2 were significantly correlated in terms of both total scores (*r* = .59, *p* < .001) and number of symptoms reported at a level of 2 or greater (*r* = .63, *p* < .001). The total number of postconcussion symptoms at both time points was significantly associated with all pain catastrophizing measures (Table 3).

Fifteen participants were classified as High Risk for developing postconcussion syndrome (6 or more symptoms at both Time 1 and Time 2) and 31 participants were classified as Low Risk. The High Risk group had significantly higher scores for PC Rumination, *t*_44_ = −2.50, *p* = .02, and overall PC, *t*_44_ = −2.28, *p* = .03, as well as a trend towards higher scores for PC Helplessness, *t*_44_ = −1.98, *p* = .05 (Table 4).

### 3.4. Pain Catastrophizing and Psychological Distress

Psychological distress (MPI Affective Distress) was positively associated with PC Rumination (*r* = .57, *p* < .001); PC Magnification (*r* = .65, *p* < .001); PC Helplessness (*r* = .67, *p* < .001); and overall PC (*r* = .66, *p* < .001).

### 3.5. Pain Catastrophizing and Level of Functionality

Level of functionality (MPI General Activities) was negatively associated with PC Rumination (*r* = −.29, *p* = .03); PC Magnification (*r* = −.43, *p* < .001); PC Helplessness (*r* = −.38, *p* = .004); and overall PC (*r* = −.37, *p* = .004).

## 4. Discussion

To our knowledge, this is the first study to investigate pain catastrophizing (PC) in the context of traumatic brain injury. Our findings suggest that PC is associated with greater severity of pain (both headache pain and general pain) in the first 8 weeks following a MTBI. This supports current evidence from various clinical settings that pain catastrophizing adversely contributes to the pain experience [30, 32, 33, 42]. Also supporting the literature, we found PC to be associated with increased psychological distress [43] and reduced level of functionality [29, 35, 42, 43]. This study also contributes a novel finding that participants with high levels of PC reported a greater number of postconcussion symptoms and had a higher risk of postconcussion syndrome development at 8 weeks after MTBI.

These results add to the compelling evidence suggesting that coping strategies, including their cognitive appraisal processes, are related to one's level of emotional distress [44, 45]. As formal diagnosis of postconcussion syndrome is not typically made until 3 months after injury [40], our results also offer an avenue for early detection of patients at risk for chronic pain and other postconcussion symptoms following MTBI. Further prospective studies are warranted to determine whether these relationships can also be observed at earlier time points. If baseline catastrophizing tendencies were shown to predict MTBI outcome, the PC scale would have considerable promise as a screening tool in the ED, given that it is of low cost and easy to administer and interpret. In turn, the early detection of PC tendencies might lead to the implementation of goal-oriented psychological interventions to prevent or minimize adverse outcomes [46]. The necessity for such tailored, case-by-case treatment interventions has been previously reinforced [47].

Our study has potential limitations. It was small in scale and conducted at a single institution. Despite compiling data on socioeconomic status, financial compensation, and work-related versus non-work-related head injury, our sample size was not sufficient to assess the potential role of these variables as predictors and/or cofactors. Further confirmatory studies are warranted, as these variables have been consistently related with slower recovery processes and unfavorable outcomes after MTBI [48, 49] and may be differentially related to pain catastrophizing tendencies. The self-reported nature of the clinical data also presents a limitation. Though evidence suggests that most patients can adequately recall numerous problematic experiences [50], the potential contribution of recall bias or selective differences in post-MTBI symptom reports cannot be overlooked [51]. Objective measurement techniques can help to address this limitation. For example, catastrophizing tendencies have been previously associated with abnormal changes to brain morphology and function, as measured on MRI [30]. A further direction of our work is to investigate the convergence of self-reported pain catastrophizing with physiological measures including brain imaging (for anatomical integrity and electrical brain activity assessment), polygraphy (for sleep and autonomic activation data), and blood sample collection (for genetic composition evaluation).

## 5. Conclusion

The present study showed that pain catastrophizing tendencies are correlated with adverse early clinical outcome in patients who sustain a MTBI. These results add to the evidence suggesting that maladaptive coping strategies are predictors of poor outcomes after traumatic injuries [43]. Moreover, our novel finding suggesting that pain catastrophizing is a risk factor for postconcussion syndrome development may facilitate early detection and intervention to prevent postconcussion syndrome. Future larger-scale studies are necessary to evaluate these factors at longer postinjury timeframes in order to optimize current MTBI treatment and improve patient outcomes.

## Figures and Tables

**Table 1:** Study population characteristics.

Demographic variables	Frequency (*N* = 58)	Total patients (%)
Occupational status		
Full-time work	44	76
Part-time work	1	2
Unemployed/searching for job	2	4
Full-time school	5	9
Retired	3	5
Seasonal work	1	2
Full-time school and part-time job	2	4
Education level		
High school	22	38
College	14	24
University	18	31
Others (vocational training)	3	5
Unknown/unspecified	1	2
Net family income ($)		
<25 000	8	14
25 000–50 000	20	35
50 001–75 000	8	14
75 001–100 000	9	16
>100 000	10	17
Unknown/unspecified	3	5

**Table 2:** Prevalence of postconcussion symptoms following mild traumatic brain injury (MTBI).

Postconcussion symptom (Rivermead Questionnaire)	Time 1^a^ (%)	Time 2^b^ (%)
Headaches	50	50
Dizziness	52	60
Nausea	16	27
Increased sensitivity to noise	25	31
Sleep disturbances	66	63
Fatigue	70	69
Irritability	30	46
Feeling depressed/teary-eyed	25	25
Feeling impatient	27	52
Forgetfulness	48	58
Reduced ability to concentrate	50	63
Slowing of thought process	30	48
Blurred vision	21	23
Increased sensitivity to light	18	25
Double vision	7	8
Feeling agitated	5	15

^a^1 month after MTBI; ^b^8 weeks after MTBI.

**Table 3:** Correlations between number of postconcussion symptoms and pain catastrophizing (PC) scores at 1 month and 8 weeks following mild traumatic brain injury (MTBI).

PC scale	Time 1^a^	Time 2^b^
Rumination	.40^*∗*^	.36^*∗*^
Magnification	.35^*∗*^	.42^*∗*^
Helplessness	.44^*∗*^	.39^*∗*^
Overall	.43^*∗*^	.41^*∗*^

^a^1 month after MTBI; ^b^8 weeks after MTBI; ^*∗*^ *p* < .01.

**Table 4:** Means (M) and standard deviations (SD) for risk of postconcussion syndrome development in relation to pain catastrophizing (PC).

PC scale	Low Risk (*N* = 31)		High Risk (*N* = 15)	
	M	SD	M	SD
Rumination	4.06	4.00	7.40	4.70
Magnification	2.00	2.08	3.07	2.22
Helplessness	4.23	4.37	7.00	4.63
Overall	10.29	9.69	17.47	10.62

## Acronyms

ED: Emergency department
GCS: Glasgow Coma Scale
MPI: Multidimensional Pain Inventory
MTBI: Mild traumatic brain injury
PC: Pain catastrophizing
RPQ: Rivermead Postconcussion Symptoms Questionnaire
TBI: Traumatic brain injury.
